# P074. Almotriptan in the acute treatment of Vestibular migraine: a retrospective study

**DOI:** 10.1186/1129-2377-16-S1-A114

**Published:** 2015-09-28

**Authors:** Domenico Cassano, Vincenzo Pizza, Vincenzo Busillo

**Affiliations:** Headache Centre, District N. 60, ASL Salerno, Nocera Inferiore, SA Italy; Headache Centre, S. Luca Hospital, ASL Salerno, Vallo della Lucania, SA Italy; Headache Centre, Maria SS. Addolorata Hospital, ASL Salerno, Eboli, SA Italy

## Introduction

Vestibular migraine (VM) has become a well-defined diagnostic entity, based on recurrent vertigo attacks, unexplained by other central or peripheral otologic abnormalities, occurring in patients with a history of migraine headache. The duration of vertigo attacks varies from seconds to days, usually lasting minutes to hours, mostly occurring independently of headaches.

## Background and objective

This was a retrospective, multicentric, open-label investigation with the aim to assess the efficacy of an oral dose of almotriptan (ALM) 12.5 mg in the treatment for acute vertigo attacks in VM, defined according to the ICHD criteria, 3^rd^ edition, beta version (2013).

This triptan, a selective 5-HT(1_B_/1_D_) receptor agonist, since its introduction in the market in 2001, has emerged as the one with the best efficacy and tolerability profile in acute migraine treatment.

## Materials and methods

The study included 26 subjects with VM (25 F, 1 M), aged from 19 to 53 years (mean, 30.0 years), reporting vertigo in more than 50% of attacks, a history of migraine for at least one year, onset of migraine before the age of 50. Three (11%) of the 26 subjects were lost to follow up; five (19%) discontinued the ALM treatment due to adverse events or any other causes.

At the time of the prescription the patients were drug-free and did not receive any prophylactic therapy.

The data were recorded in a headache diary: the intensity of vertigo attacks was assessed by a 3-score scale (with “1” indicating mild vertigo, “2” medium intensity and “3” the worst vertigo imaginable), while the therapeutic response to vertigo attacks by a 4-score scale (with “0” indicating any change, “1” under 50% reduction, “2” over 50% reduction and “3” the complete disappearance of vertigo). Almotriptan was administered as a single 12.5 mg tablet with the advise to take the drug within 1 h of onset of vertigo attack.

Follow-up was performed every month for the following three months after the ALM treatment initiation and the response on vertigo attacks during the study were considered as the main and primary outcome, while secondary variable was the effect on pain relief at 2 and 4 hours.

From the first visit onward, patients reported if they had experienced any adverse events.

Statistical analysis of data was carried out using student t-test.

## Results

Eighteen patients were examined; they reported 27 vertigo attacks in the course of the three-month follow-up, with mean intensity scores ranging between “2” (24%) and “3” (76%).

Among all the patients, 10 (55%) reported complete disappearance of vertigo, 5 (28 %) over 50% reduction and 3 (16%) under 50% reduction. Also, the pain relief were significantly reduced since the first month and confirmed in the following two months (p < 0.001).

These data suggest a benefit from almotriptan at the oral dose of 12.5 mg in 83% of the patients with vestibular migraine attacks; good was the tolerability profile.

## Conclusions

This study suggests that almotriptan is effective and safe in reducing both vertigo and headache among patients who suffer from Vestibular migraine. This will have to be reconfirmed in a large scale, randomized, controlled clinical trial.

Written informed consent to publication was obtained from the patient(s).

